# Experiences Using a Multidisciplinary Model for Treating Injection Drug Use Associated Infections: A Qualitative Study

**DOI:** 10.3389/fpsyt.2022.924672

**Published:** 2022-06-21

**Authors:** Nathanial S. Nolan, Emily Gleason, Laura R. Marks, Tracey Habrock-Bach, Stephen Y. Liang, Michael J. Durkin

**Affiliations:** ^1^Department of Medicine, Division of Infectious Diseases, St. Louis School of Medicine, Washington University, St. Louis, MO, United States; ^2^Perelman School of Medicine, University of Pennsylvania, Philadelphia, PA, United States; ^3^Department of Emergency Medicine, St. Louis School of Medicine, Washington University, St. Louis, MO, United States

**Keywords:** persons who inject drugs, opioid use disorder, substance use disorder, AMA discharge, medications for opioid use disorder

## Abstract

**Background:**

Over the past two decades, the United States has experienced a dramatic increase in the rate of injection drug use, injection associated infections, and overdose mortality. A hospital-based program for treating opioid use disorder in people who inject drugs presenting with invasive infections was initiated at an academic tertiary care center in 2020. The goal of this program was to improve care outcomes, enhance patient experiences, and facilitate transition from the hospital to longer term addiction care. The purpose of this study was to interview two cohorts of patients, those admitted before vs. after initiation of this program, to understand the program's impact on care from the patient's perspective and explore ways in which the program could be improved.

**Methods:**

Thirty patients admitted to the hospital with infectious complications of injection drug use were interviewed using a semi-structured format. Interviews were transcribed and coded. Emergent themes were reported. Limited descriptive statistics were reported based on chart review.

**Results:**

Thirty interviews were completed; 16 participants were part of the program (admitted after program implementation) while 14 were not participants (admitted prior to implementation). Common themes associated with hospitalization included inadequate pain control, access to medications for opioid use disorder (MOUD), loss of freedom, stigma from healthcare personnel, and benefits of having an interprofessional team. Participants in the program were more likely to report adequate pain control and access to MOUD and many cited benefits from receiving care from an interprofessional team.

**Conclusions:**

Patients with opioid use disorder admitted with injection related infections reported improved experiences when receiving care from an interprofessional team focused on their addiction. However, perceived stigma from healthcare personnel and loss of freedom related to hospitalization were continued barriers to care before and after implementation of this program.

## Introduction

Over the past two decades, the United States (U.S.) has experienced a dramatic increase in misuse of both prescription and non-prescription opioids. A three-phase epidemic, which started with prescription opioids and progressed to illicit heroin and then fentanyl, has now culminated in a dramatic rise in injection drug use (IDU) across the U.S. (1). In the last year, overdose deaths have rose to over 100,000, with over 60% involving synthetic opioids (2). Complications related to IDU have also increased (3). People who inject drugs face higher rates of serious bacterial, fungal and viral infections (specifically human immunodeficiency virus and viral hepatitis) (4–7).

A growing body of evidence suggests that hospital outcomes for infectious complications of IDU are improved when people who inject opioids are treated with medications for opioid use disorder (MOUD) (8–11). In patients presenting with OUD, MOUD have been associated with a decrease in all-cause mortality, overdose events, and need for acute care related to opioid use (12, 13). However, initiation of MOUD may not be enough to improve outcomes; patients not remaining on these medications lose the survival benefit previously imparted by them (14, 15). Unfortunately, many patients with OUD who are discharged from the hospital struggle to find access to MOUD. Large organizations, including the National Academies of Sciences, Engineering, and Medicine, have called for more resources to increase MOUD prescribing and the development of programs to link patients to community-based treatment (16). A continuum of care model, similar to that used for patients living with HIV, has been proposed with the goal of transitioning patients along a care pathway, from identification, to stabilization, and linkage to long-term OUD management (17, 18).

The Washington University School of Medicine bridge-to-health program was initiated as a Centers for Disease Control and Prevention (CDC) funded program for treating opioid use disorder (OUD) in people who inject drugs (PWID) presenting to the hospital with invasive infections associated with injection drug use (19). Patients that are prospectively identified by infectious disease physicians, hospitalists, and social workers can be enrolled in the program, which provides access to a peer recovery coach, a dedicated program social worker, a clinical counselor, and physicians who can follow-up post discharge. Participants receive free MOUD, vaccinations to prevent injection associated infections (e.g., hepatitis A and B), and linkage to post-discharge infectious diseases care that can provide oral antibiotics for patients who discharge prior to completion of IV antibiotics, pre-exposure prophylaxis for HIV, and treatment for hepatitis C infection (when appropriate). If patients continue using injection drugs, they are also offered a harm reduction kit that includes wound care supplies, alcohol swabs, and educational materials. All patients are offered take-home naloxone. Patient enrollment in the program is voluntary and requires no long-term commitment. Upon hospital discharge, patients are followed in a bridge-to-health clinic, either in-person or via telemedicine, and continue to receive intensive social work and peer recovery resources for 90 days after discharge, followed by a handoff to community providers for continuation of their addiction care. The goal of this program is to provide supportive services for PWID presenting with injection related infections, decrease readmissions, and improve retention in outpatient infectious diseases and substance use disorder care (19).

The purpose of this study was to interview patients admitted to the hospital with a serious injection-related bacterial or fungal infection before and after initiation of the multidisciplinary bridge-to-health program, to understand its impact from the patient's perspective and explore ways in which the program could be improved.

## Methods

From April to October 2020, we conducted 30 semi-structured interviews with patients admitted to a 1400-bed academic, tertiary hospital in St. Louis, Missouri, for invasive infections related to IDU. Adults, over the age of 18 years, hospitalized for a serious IDU-related infection (i.e., endocarditis, osteomyelitis, septic arthritis, epidural abscess or *S. aureus* bacteremia) from January 2018 until October 2020 were eligible for participation. There were no exclusion criteria. Eligible patients were recruited, via phone call, from a cohort of patients who were being treated and followed in an infectious disease clinic for infections related to their drug use. Interviewees provided informed verbal consent and were given a $20 gift card for their participation. This study was approved by the Washington University Human Research Protection Office.

Each patient who consented to partake in this study participated in a phone interview (ranging from 10 to 40 minutes) with one of three research assistants (two male, one female) trained in qualitative interviewing. These research assistants were peer recovery coaches for the bridge-to-health program who often developed a rapport with patients through the context of the program. Topics included (1) nature of the patient's hospital experience and interactions with staff; (2) desire to leave against medical advice (AMA); (3) motivation and resources for recovery from OUD (e.g., MOUD, social support, abstinence-based groups); (4) self-management of past skin or minor infections; (5) knowledge and use of practices to prevent infection (e.g., HIV pre-exposure prophylaxis, hepatitis vaccination, use of sterile needles); and (6) feedback to healthcare personnel on how best to support patients with OUD. Questions targeted the experiences patients had while hospitalized and no questions specifically asked about patient experiences in the bridge-to-health program. Study members recorded and transcribed the interviews. Quantitative data were abstracted from the medical record with permission from the participants.

Interview transcripts were coded thematically using NVivo qualitative research software (NVivo 12, QSR International). A constructivist grounded theory approach was taken, with the goal of exploring the experiences of two cohorts of patients admitted to the hospital with OUD: those admitted before and those admitted after the initiation of the bridge-to-health program (20, 21). Reviewers were blinded to whether the study participants were engaged in the program. NN, an infectious disease fellow, and EG, a pre-medical student with a background in qualitative research, independently reviewed each audio recording and transcript. The reviewers corrected for transcription errors or omissions. Codes were generated inductively using an open coding process with active comparison between coders. Axial coding was used to generate a codebook, with iterative modification of the codebook based on re-review of the transcripts. These two researchers then independently coded all 30 transcripts and discussed any coding ambiguities or discrepancies. Codes were diagramed according to emergent themes and were analyzed based on time period, specifically pre- vs. post-implementation of the bridge-to-health program. Illustrative quotes were extracted to facilitate presentation of the data. All methods are reported according to established best practices (22).

## Results

Thirty study participants were interviewed about their experiences being hospitalized for IDU-related bacterial or fungal infections. Just under half (*N* = 14) of the participants were hospitalized prior to initiation of the bridge-to-health program (which started in February 2020). Most participants (*N* = 18; 60%) were men. The average participant age was 41.5 years old (SD ± 11.4). The average length of stay was 25.5 days (SD ± 21.3). Nine patients left AMA prior to completion of their hospital care (6 in the post intervention group). Nineteen participants had an addiction medicine consult, with fewer consults received during the preintervention period (5/14) compared with the post-intervention period (14/16). Twenty-two participants were prescribed MOUD at discharge (15 prescribed buprenorphine-naloxone, 4 prescribed methadone). The most common IDU-related infections were endocarditis (*N* = 13), osteomyelitis (*N* = 10), and complicated skin and soft tissue infections (*N* = 10) (some patients had more than one infection). Basic demographics are presented in Table 1.

**Table 1:** Participant characteristics.

	**Pre**	**Post**	***p*-value**
	***N* = 14**	***N* = 16**	
**Demographics**			
Age (mean, SD)	40.4 (10)	42.5 (11)	
Male	8 (57.1%)	10 (62.5%)	0.77
Female^&^	5 (35.7%)	6 (37.5%)	0.92
Transgender	1 (7.1%)	0 (0%)	0.21
White	4 (28.6%)	8 (50.0%)	0.38
Homeless	2 (14.3%)	3 (18.8%)	0.81
Rural County	3 (21.4%)	4 (25.0%)	0.91
Substance use patterns*			
Heroin	13 (92.9%)	13 (81.3%)	0.34
Fentanyl	5 (35.7%)	10 (62.5%)	0.12
Methamphetamine	3 (21.4%)	5 (31.3%)	0.54
Cocaine	5 (35.7%)	4 (25.0%)	0.52
**Inpatient characteristics**			
MOUD initiated	8 (57.1%)	14 (87.5%)	0.05
Addiction Medicine consult	5 (35.7%)	14 (87.5%)	<0.01
Reason for admission*			
Infective endocarditis	8 (57.1%)	5 (31.3%)	0.15
Osteoarticular Infection	5 (35.7%)	7 (43.8%)	0.65
Complicated skin and soft tissue Infection	4 (28.6%)	5 (31.3%)	0.87
**Comorbidities**			
Hepatitis C	9 (64.3%)	10 (62.5%)	0.92
Human Immunodeficiency Virus	1 (7.1%)	2 (13.3%)	0.62

*^&^One female was transgender*.

^*^Patients may use more than one type of substance, and may have multiple concurrent infectious complications.

### Pain Control and Access to MOUD

When exploring patient narratives, it became clear that many had past traumas associated with inadequate pain control, which frequently impacted how they interacted with their healthcare teams. When discussing a prior hospitalization, a 40–50-year-old White female said, “I felt like they thought, because I was an addict, that I deserved to in the pain I was in.” These experiences made patients reluctant to seek care and, when patients did seek care, pain was frequently a source of conflict between them and their hospital team. Even when pain was not a pressing concern, others reported being forced to withdraw without the option of opioid replacement therapies. A 20–30-year-old Black woman, who used opioids and was hospitalized prior to implementation of the bridge-to-health program, explained:

“*When people come in on drugs with withdrawals and everything, they don't be so quick to get you methadone… I feel that if a person comes in sick with an infection and is on drugs… they should have an option whether they want to be put on any type of methadone or some type of Suboxone [buprenorphine/naloxone] or just want to withdrawal on their own… That's a lot of why I left, because I'm like, ‘I'm not going to sit here dope sick.”'*

Several other participants noted that withdrawal and cravings were a significant reason for leaving against the advice of their medical providers.

Participants who were able to participate in the bridge-to-health program often reported improved pain control as compared to prior experiences. For example, when asked about her experience, a 30–40-year-old White female, who used heroin and participated in the program, stated, “they had me on painkillers, because when I was in the hospital, I was in there for surgeries. So, it wasn't so bad. And then when I came down, they gave me Suboxone.” Withdrawal and cravings were less likely to be noted following implementation of the program, particularly in those who received addiction medicine consultations. When withdrawal was brought up, it was most often as a discussion of prior experiences. One 30-40-year-old Black male, admitted to the hospital following implementation of the bridge-to-health program, reported that his most recent hospital stay “wasn't that bad [because] they treated my withdrawals.” Further illustrative quotes for all themes can be found in Table 2.

**Table 2:** Themes and quotes from qualitative interviews.

**Theme**	**Quotes**
Pain control and access to MOUD	They should be getting people on Suboxone. They should be setting that stuff up prior before they leave the hospital. - *30–40-year-old White male. Not a participant in bridge-to-health program* The doctors were either awesome or they were very callous. I don't think the doctors respected the fact that I was an addict. And just because you give somebody a prescription and tell them to just take it a certain way, does not mean that individual's capable of it. And one mistake on my part could put a needle back in my arm. And I just feel, after a certain point that they just wanted to... I don't understand why it was so difficult for me to get on, stay on, and be put back on the Suboxone. *- 20–30-year-old Black male. Participant in bridge-to-health program*
Stigmatization	I mean, they were all pretty good. It was just that first doctor, like I said, it felt like he was judging me the whole time and I was restricted on some things when other patients I would talk to, or whatever, they had these liberties or whatnot that I didn't have at the time. It was because he knew that I was a user. And I don't think that really should have mattered, whether I do or not. I should have been treated the same regardless. And if I did start abusing something, then, take action. But if I'm not doing anything wrong, who cares? I should be treated the same way as everybody else. *- 30–40-year-old White male. Not a participant in bridge-to-health program* I had doctors tell me, ”I don't care, leave.“ They're just gonna go back and use to get high and die ... I'm talking about, this is the doctor telling me that. They're saying to me and that's the person they got to worry about and I'm sitting there like, ”What?" We don't need to hear that. *- 20–30-year-old Black female. Not a participant in bridge-to-health program*
Loss of freedom	“It'd have been nice if they had somebody come around, maybe once a week, when people were able to get up on their own safely, and be able to go outside and maybe get fresh air… not being able to go outside… that was the hardest part.” *- 50–60-year-old White male. Not a participant in bridge-to-health program*
Person-centered care	This one nurse, [xxxx] was her name, she would come in, she would make time every night to come in, because I couldn't take a shower for a while ... I couldn't get in the actual shower. But she would make time every night to come help me wash my hair at the sink. She shaved my legs for me. She hand-washed some of my clothes that I had. I mean, it just made me feel really good, and I know she wasn't doing it for recognition. I could tell she was just doing it because she cared, and she liked her job, and I really thought that was amazing. *- 40–50-year-old White female. Participant in bridge-to-health program*
Harm reduction	Because they make it hard so where you can't get clean needles. I mean, I never really had a difficult time, because I knew what stores to go to, but I know it is hard for people that don't know where the stores are, because a lot of places won't sell you clean needles unless you are on insulin. Which I mean, I understand they're trying to cut down, but in a way if a person wants to get high, they're going to get high. So why not let them be able to use clean utensils rather than spread disease? Because they're going to do it regardless. *- 40–50-year-old White female. Participant in bridge-to-health program*
Benefits of a Multi-disciplinary bridge model	When you're not feeling judged, then you're willing to hear all the options that they have for help, and I really think that's the most important thing is offering the help and options for when they go home. What helped me the most is being able to have somebody like [my recovery coach] that I can talk to about any problems, or cravings, or anything. And then having [my social worker] who I can ask for any help I need help with as far as a case worker. And [my doctor], I mean, she calls me just to check on me. That made me feel so important and special. *- 40–50-year-old White female. Participant in bridge-to-health program*.

### Stigmatization

Many participants described barriers to care beyond pain control, including interpersonal conflicts with clinicians and the experience of judgement or stigma. Even with adequate pain control or the appropriate prescription of MOUD, these additional factors contributed to poor hospital experiences and AMA discharge in some instances. Measures such as direct patient observation (i.e., patient sitters), inability to leave the unit, and searches by security underscored the lack of trust on the part of the clinicians and created a more hostile environment. One 30–40-year-old White male explained, “it felt like [the doctor] was judging me the whole time and I was restricted on some things when other patients I would talk to, or whatever, they had these liberties, or whatnot, that I didn't have at the time.”

Some participants had poor interactions with specific members of the healthcare team. One participant recounted how she was denied the antiemetic promethazine by one clinician because it had abuse potential. These episodes reiterated how participants with history of addiction were “othered” in the hospital. A few participants who chose to leave against medical advice cited a single episode of conflict as the inciting factor. When discussing an interaction with a nurse practitioner, a 30–40-year-old White female participant explained:

“*She ended up coming and wanting to search the room, which was no problem. I had nothing in the room. But I felt like after they searched the room and didn't find anything, and they searched my boyfriend… and didn't find anything… They were still going to make somebody sit there and like babysit me… I just felt kind of disrespected.”*

This negative interaction with a single provider led the participant to discharge against medical advice. “That's ultimately why I left,” she elaborated, “because of how the nurse practitioner [treated me], I felt like she was singling me out.”

When analyzing the experiences of participants before and after initiation of the bridge-to-health program, we found that episodes of stigma and judgement continued to exist post-implementation. However, distinctions emerged, suggesting improved overall experience. Participants in the post-implementation group frequently contrasted negative experiences in other clinical settings with the positive experiences in the bridge-to-health program. For example, a 40–50-year-old White female described:

“*My interactions during [this hospitalization], the doctors and the nurses, they were great. I believe they did everything really to their abilities to try and help me, and they did not make me feel like I was any less because I was an addict.”*

Despite the program, patients continued to suffer stigmatization, however these concerns were less frequently cited in the cohort of patients admitted to the bridge-to-health program.

### Loss of Freedom

Confinement and lack of freedom were frequently brought up by participants, particularly because many patients who use drugs require prolonged hospital stays to receive intravenous antibiotics. A 20–30-year-old Black male who used multiple substances, including opioids and methamphetamine, said, “I had to get used to not being able to come and go as I please. I used to eat what I want, to sleep when I want, or to roll over in bed at two o'clock in the morning and light up a cigarette.” For him, even with access to addiction care and peer recovery coaches, loss of freedom made it difficult to stay in the hospital for a prolonged period of time.

The coronavirus disease 2019 (COVID-19) pandemic occurred during the time of this study, resulting in new policies limiting hospital visitors and discouraging patients from leaving their rooms or congregating in common areas. Many participants discussed how changes in hospital policies impacted their care. A 40–50-year-old White female who left AMA explained, “I was fighting drug addiction, but it was being alone. I don't like to be alone. I don't like to be alone… I have abandonment issues.” For some of these participants, loss of hospital visits and uncertainty at home complicated their care experience. The theme of COVID-19 and its impact on these patients was recently reported by this team (23).

Unfortunately, the addition of our focused, interdisciplinary team did not alleviate the feeling of isolation and confinement described by many study participants. Though they were thankful for regular visits by peer recovery coaches, there was a profound sense that their freedom was being impacted. Even the fact that their treatment required prolonged hospitalization was enough to make them feel different from other patients. Further, patient obligations often persisted during their hospitalization. One 40–50-year-old White male provided his reasoning for leaving his hospital stay early, “I was ready to get back home because my life was at home and I just got dragged out of it. That's the only reason I left… I missed my family and my family is everything to me.” He went on to describe family commitments as the driving force for a discharge against his medical team's advice.

### Person-Centered Care

Many participants were quick to highlight the positive aspects of their hospital experiences, particularly physicians or nurses who stood out as exceptional. Even small acts of kindness secured good will and improved the patient's overall view of the staff and healthcare team. A 30–40-year-old White male told the story of coming out of surgery late and missing his dinner tray, “one of the nursing staff members, I can't remember his name, he went and bought me supper down at the cafeteria, with his own personal money.” This act left a clear impression on the patient. Another participant, a 20–30-year-old Black male, described how the hospital staff rallied behind him and became a new surrogate support system. “I wanted to be dead... and this woman, this doctor, she helped me through it.” He described hospital staff visiting him on their off days and calling to check in on him. Another participant, a 40–50-year-old male, explained how his stay was improved by the excellent nursing staff, “they treated me like family. I was a long way from home and I didn't have no family there with me or nothing. They made that stay better.”

### Harm Reduction

Participants were asked about existing personal practices to prevent infection and how these could be better supported. Participants often brought up the idea of needle exchanges and safe injection sites, which were not legal in Missouri at the time the study was performed, with the closest needle exchange locations for participants being in neighboring states, such as Illinois. A 40–50-year-old White female explained that her friend lived in a location with safe injection sites: “I can't imagine going somewhere and having somebody help me do it. But at the same time, if there was somebody that could... I think about how my body wouldn't look like it is right now.”

Many participants had a strong understanding of safer injection practices; they frequently cited the experience of being admitted with an invasive IDU-related infection as a life-altering event. A 40–50-year-old White woman admitted with endocarditis from injection fentanyl use described her practices regarding injection preparation:

“*I just do everything as possibly clean as I can. I mean, the water, all of it, just because I know how easy something can happen. And you can think, just like I thought when that happened, that I was doing everything right. I wasn't dirty, but there was something that happened to it. So, yeah. I do things a lot different than I did at that time, a lot cleaner and I won't use a needle more than one time.”*

Though many patients expressed understanding of safe injection practices, gaps in understanding regarding medication and vaccine prophylaxis continued to persist, even after the bridge-to-health program initiation. Many participants had little understanding about HIV pre-exposure prophylaxis, which was described by this group elsewhere (24). Further, many had little knowledge about their vaccination status for Hepatitis A and Hepatitis B. Even when records demonstrated that patients were vaccinated during their hospitalization, this frequently was not recalled.

### Benefits of a Multi-Disciplinary Bridge Model

Overall, those who were able to participate in the bridge-to-health program were appreciative of the services and frequently cited the benefit of a multidisciplinary approach. A 40–50-year-old White female, who participated stated:

“*What helped me the most is being able to have somebody like [my recovery coach] that I can talk to about any problems, or cravings, or anything. And then having [my social worker] who I can ask for any help I need with as far as a case worker. And [my doctor], I mean, she calls me just to check on me. That made me feel so important and special.”*

Similarly, a 60–70-year-old White male, said, “I have my coach here… I have the methadone clinic, suboxone that I could try. I mean the whole team here is awesome. The doctors, the nurses here, everybody who's involved in my treatment has been awesome.”

Participants who were not part of the bridge-to-health program often spoke of their care being disjointed or lacking resources. For example, when a 30–40-year-old White female was asked if she had the resources to quit opioids she responded:

“*I do not. Okay. That kind of a question has two different answers to it, because I do have a great support system. My family that will be, and is trying to be, behind me, and all of those different things. But I've never been able to just go through quitting cold turkey on my own.”*

She elaborated that her struggles with anxiety and not having adequate medications for her addiction have further prevented recovery. Many explained that upon discharge from the hospital they were unable to find stable addiction care or access to medications to treat their addiction. The rate of return to drug use was high in the cohort of patients prior to implementation of the bridge-to-health program.

## Discussion

This qualitative analysis was undertaken to explore patient perceptions of a multidisciplinary program to address opioid use disorder (OUD) in the context of hospitalization for a serious injection-related infection. This program (called the bridge-to-health program) was designed to bridge the gap between hospitalization, early discharge and establishment of stable addiction care (19). When comparing interviews with patients admitted before and patients admitted after the initiation of this program, we found several perceived benefits, as well as areas of improvement.

Perhaps the most significant in-hospital change offered by the bridge-to-health program were improvements in access to medications for OUD (MOUD) and overall pain control. Inadequate pain control is often a concern for patients with tolerance to opioids. Patients struggling with addiction have reported being denied analgesia due to concerns that they are “drug-seeking” (25, 26). When analgesia is provided, it may be inadequate due to increased opioid tolerance. Participants in our study had similar concerns. However, following implementation of the bridge-to-health program, we noted fewer concerns about inadequate pain control. In the bridge-to-health cohort, addiction services were part of the program and frequently helped patients and their medical teams navigate acute opioid needs along with initiation of MOUD, when indicated. We found that early involvement of addiction specialists led to one of two outcomes: early initiation of MOUD or recommendation to continue short-acting opioids for pain and delaying initiation of MOUD until the patient was more stable, with a smoother transition off acute opioids and onto MOUD. Lack of addiction medicine consults have been associated with inadequate access to MOUD, even when interprofessional care teams are assembled to improve addiction care (27). Previous data has found that patients with OUD hospitalized with injection-related infections and treated with MOUD have improved outcomes and are less likely to leave AMA (9, 11). Similarly, addiction medicine consults improve patient outcomes (9). These patient narrative data suggest that treating OUD during the hospital stay helped to make the hospital stay less traumatic.

Our results echo other researchers' findings that patient stigmatization by healthcare personnel remains a substantial barrier to improving the care of PWID (10, 28, 29). It is notable that Pollini and colleagues describe an almost identical story to one described above, of a patient having their belongings searched and experiencing inappropriate scrutiny, which ultimately led to an early, patient-directed discharge (30). For many participants in our study, the experience of significant stigma seemingly left the mark of lasting trauma, coloring their interactions with healthcare professionals moving forward. Our qualitative approach adds to the literature by providing several vivid examples, from the patient's perspective, of how stigma harms healthcare interactions and leads patients to leave AMA, or avoid accessing care altogether (31).

However, our findings also identified that reducing stigma can improve healthcare personnel interactions with PWID. In particular, small gestures of goodwill and other displays of person-centered care made a lasting impression on patients and improved the overall patient perception of the hospital experience. At this time, it is unclear what interventions might directly reduce patient-experienced stigma, however we hypothesize that normalization of addiction care through the use of interprofessional teams can help to drive institutional cultural change. Anecdotally, when peer-recovery coaches were added to healthcare teams, all of whom had previously recovered from addiction, important perspectives were added to the care team discussion. Partnering and working with those who have lived experience of the stigmatized condition has been used as a destigmatizing process and may help physicians, nurses, and other health professionals see PWID differently (32, 33).

In our study, feelings of confinement and lack of freedom were common. Hospital policies restricting the movement of PWID, particularly during the COVID-19 pandemic, likely played a role in these perceptions. These concerns were frequently identified among patients who required prolonged inpatient stays for intravenous antibiotics and wound care. These feelings were not alleviated, even with the addition of the bridge-to-health program staff, and were associated with early, patient directed discharge. Others have noted that isolation, loneliness and boredom are associated with difficulty staying in the hospital to complete long courses of treatment (30). Further research is needed to determine how to best reduce these feelings of confinement, isolation, and boredom among patients.

Much of the bridge-to-health model is centered on harm reduction principles. During initial visits, patients are educated on practices that might have been associated with infection, such as re-using or licking needles, using non-sterile water, and failing to sterilize the skin. While the goal is to help patients avoid injection drug use, those who continue to use are offered education and supplies for infection prevention. While most participants endorsed a basic understanding of infection prevention principles, many cited a lack of support in the surrounding community (e.g., lack of needle-exchanges, lack of safe injection spaces, lack of safe syringe disposal, etc.). Despite education, participants had limited knowledge regarding vaccine and medication strategies as infection prevention. Future efforts will be aimed at expanding education, access and patient support.

Participants who were able to enroll in the bridge-to-health program reported benefits to having a dedicated team that spanned their treatment both inside and outside of the hospital. In this study, more patients who left against their medical team's advice were included in the post intervention analysis. While this might seem curious, it is because, despite leaving against medical advice, these participants remained reachable, often providing reliable contact information and agreeing to follow up before leaving the medical facility. Further, the involvement of a dedicated, interprofessional team focused on co-management of substance use disorder and infections aided discharge discussions, similar to that proposed by other models (34). In contrast, preintervention patients were left without similar options, and many who did discharge against the medical team's advice did so in a less coordinated way, often remaining unreachable or, when reached, declining to discuss their stay.

Interprofessional care teams, similar to the model described in this study, are emerging tools to help combat the opioid epidemic (35). However, some have reported limited success in helping patients to start and remain on medications to treat their opioid use disorder (27). We found that this bridge model, comprising a team that followed the patient in the hospital, and their transition out of it, helped to engage patients and increased retention in follow up care.

### Limitations

This study is subject to several important limitations. First, interviews were performed by peer recovery coaches. This increases the risk of acquiescence bias. However, even patients enrolled in the bridge-to-health program seemed uninhibited in sharing negative viewpoints about their hospital care. Further, the interview was very specific in asking about patient's experiences with nurses, staff, etc. It did not ask about patient experiences with the bridge-to-health program or any of its staff. The interviews were not mandatory and played no role on participation in the program. As is typical, sampling bias may have occurred based on those willing to participate in study. Another significant bias is that of recall. Participants in the bridge-to-health program would have been more recently hospitalized, meaning their recall may be clearer, which likely limits some ability to draw comparisons. Some of our patient experiences, particularly those related to confinement and lack of freedom in the hospital, could have been confounded by stricter hospital visitor policies during the COVID-19 pandemic. However, our team also observed this theme among patients hospitalized preceding the pandemic. Finally, the bridge-to-health program grew organically, and quality improvement efforts aimed at improving care for patients admitted with invasive infections related to their addiction had been ongoing before the formal program was established. Some pre-implementation participants may have benefited from services similar to those provided later by the program.

## Conclusions

In the past two decades there has been a dramatic increase in the number of patients presenting to hospitals for complications of injection drug use. Caring for these patients can be difficult, as physicians attempt to balance acute needs with the treatment of the patient's underlying drug use disorder. Further, these patients are at high risk for recidivism and loss to follow up when they transition out of the hospital. A multi-disciplinary model, named the bridge-to-health program, helped to improve the care experiences of patients admitted with infectious complications of their injection drug use. This program improved experiences around pain control, withdrawal, and navigation of care following hospitalization. However, much work still needs to occur when managing stigma and patient loss of freedom (due to hospitalization). This data will inform further quality improvements in the bridge-to-health model and serves to demonstrate its benefits from the patient's own perspective.

## Data Availability Statement

The datasets presented in this article are not readily available because they are qualitative data that include personal patient interviews. They are stored in a HIPAA compliant manner and as part of consent, we agreed not to share the full interview. Further enquiries can be directed toward the corresponding author.

## Ethics Statement

The studies involving human participants were reviewed and approved by The Washington University in St. Louis Institutional Review Board. The patients/participants provided their recorded verbal informed consent to participate in this study.

## Author Contributions

NN and EG performed analysis of the transcribed interviews and wrote the initial drafts of this manuscript. LM, TH-B, SL, and MD were important in creating the interview questions, evaluating data, and providing critical feedback on the final draft of the manuscript. All authors had access to the data and a role in writing the manuscript.

## Funding

This work was supported by the CDC via a Safety and Healthcare Epidemiology Prevention Research Development (SHEPheRD) Contract number 200-2016-91804 order numbers 75D30119F0001 and 75D30119F00002. MJD receives salary support from the National Institutes of Health, under award numbers K23DE029514 and R21DA053710. LM was supported by the National Institutes of Health, under award number T32AI007172. SL received support through the Foundation for Barnes-Jewish Hospital and the Washington University Institute of Clinical and Translational Sciences which is, in part, supported by the NIH/National Center for Advancing Translational Sciences (NCATS), Clinical and Translational Science Award (CTSA) program (UL1TR002345).

## Conflict of Interest

The authors declare that the research was conducted in the absence of any commercial or financial relationships that could be construed as a potential conflict of interest.

## Publisher's Note

All claims expressed in this article are solely those of the authors and do not necessarily represent those of their affiliated organizations, or those of the publisher, the editors and the reviewers. Any product that may be evaluated in this article, or claim that may be made by its manufacturer, is not guaranteed or endorsed by the publisher.
